# Creating a Healthy Built Environment for Diabetic Patients: The Case Study of the Eastern Province of the Kingdom of Saudi Arabia

**DOI:** 10.5539/gjhs.v6n4p136

**Published:** 2014-04-14

**Authors:** Bhzad Sidawi, Mohamed Taha Alhariri, Walid Ibrahim Albaker

**Affiliations:** 1 College of Architecture and Planning, University of Dammam, Kingdom of Saudi Arabia; 2 Department of Physiology, College of Medicine, University of Dammam, Kingdom of Saudi Arabia; 3 Department of Endocrine and internal medicine, College of Medicine, University of Dammam, Kingdom of Saudi Arabia

**Keywords:** unhealthy built environment, diabetes symptoms, perception of place

## Abstract

Many studies worldwide have demonstrated the negative impact of an unhealthy built environment on citizens. In the case of diabetes, studies have concentrated on the environmental impact and accessibility issues of a place i.e. the home and neighborhood, whereas few studies have addressed the comfort of the type and spatial arrangement of a household and linked it with the prevalence of diabetes. Also, little research has tackled the place’s impact on diabetic patients and their views concerning their environments. This paper demonstrates the outcomes of survey that was carried out on diabetic individuals who usually visit the King Fahd teaching hospital of the University of Dammam, Al-Khober, the Kingdom of Saudi Arabia (KSA). The patients were surveyed and physically examined. The present researchers found significant links between patients’ diabetes symptoms such as reported paresthesia and blurred vision, and medical investigations results such as lipid profile, blood glucose and blood pressure with the environmental conditions of their homes and neighborhoods. The paper shows that the prevalence of the disease is not only caused by an unhealthy lifestyle but also by an unhealthy built environment. Moreover, it illustrates that unhealthy built environment promotes unhealthy life styles. It makes recommendations on how to improve the built environment in the KSA to be healthier for all citizens including the diabetic patients.

## 1. Introduction

The provision of a healthy built environment became increasingly under focus nowadays as profound links are found between a healthy built environment and healthy lifestyles. The design of a healthy environment would facilitate social cohesion and offer access to educational, cultural, leisure and retail facilities capable of sustaining urban development (Barton, Grant, & Guise, 2010; Barton & Grant, 2011). Research suggests that diabetes is caused by a complex interaction between patient’s genetics and environment factors. Barton and Grant (2011) suggested that, nowhere is the critical nature of the relationship between the built environment design, healthy citizens, and lifestyles better illustrated than when considering chronic diseases such as diabetes.

Barton et al. (2010) pointed out to the direct and indirect impact of the built environment on health. Among the indirect effects are the place i.e. Home and neighborhood characteristics and the people’s perception of their neighborhoods. In the case of Kingdom of Saudi Arabia (KSA), certain cultural, environmental, and urban constraints would affect health. The consideration of such factors, would not only tackle the tragic spread of “*unhealthy lifestyles*”, and enable the built environment to contribute to the healthy lifestyles of diabetic patients in the KSA but also sustain the healthy urban development of towns and cities in the KSA. This research investigates the possible indirect built environment and spatial arrangement impacts on the health of diabetic individuals in the KSA. A survey has been carried out on a sample that consists of seventy six patients who usually visit the King Fahd teaching hospital of the University of Dammam, Al-Khober. The patients were surveyed and physically examined. The field study’s results are discussed and linked with previous research studies.

The next section below discusses the diabetes symptoms and complications and the occurrence of the disease in developed countries including the KSA. Section three and four demonstrate the adverse impacts of the built environment on people, and section five introduces the characteristics of the healthy built environment. Section six highlights the research methodology and tools. Section seven outlines the survey results, and discusses the statistical links between the physiological conditions of the patients with their homes and neighborhood settings and their perception of the neighborhood’s conditions, thus section eight suggests future research venues.

## 2. The Diabetes Symptoms and Its’ Prevalence in the KSA

Diabetes mellitus is the most common non-communicable disease worldwide and the fourth to fifth leading cause of death in developed countries. There are – in general- two types of diabetes. In Diabetes type I (T1DM), the person’s own body has destroyed the insulin-producing beta cells in the pancreas. Although type II diabetes mellitus (T2DM) can be caused by genetic factors, an unhealthy lifestyle happens to be the main cause (Sidawi & Hariri, 2012). A person with T2DM has one of two problems, and occasionally both: a) not enough insulin is being produced; and b) the insulin is not working properly. The most common early warning signs include: frequent need to urinate; increase in thirst; rapid weight loss; blurry vision; rash or itching skin that can result in an infection; frequent tingling sensation in the hands and feet; and fatigue. While the direct symptoms of diabetes, such as thirst, frequent urination and fatigue, can be mild and may cause little interruption to activities of daily living, it is the complications of the disease, including blindness in adults (Jeppesen & Bek, 2004) non-traumatic lower-limb amputation (Chaturvedi, Stevens, Fuller, Lee, & Lu, 2001) and kidney failure that result in transplantation and dialysis (Atkins, 2005). In addition, the risk of coronary heart disease is two to four times higher in diabetic patients. The risk of stroke or peripheral vascular disease also increases strongly. In fact, the management and treatment of diabetes mellitus, mainly T2DM, is considered to be more than the mere control of blood glucose values, asking for a multidisciplinary approach (i.e. shared care) to reduce macro- and micro-vascular risk factors (American Diabetes Association, 2006). The Kingdom of Saudi Arabia has one of the highest percentages of diabetes in the world, with an estimated number of 3,414,510 people diagnosed with the disease in 2012, which is 23.38% of the population (International Diabetes Federation, 2012).

Previous surveys from the KSA suggested that diabetes is present in epidemic proportions throughout the country with exceedingly high rates concentrated in urban areas (Alzaid, 1997). Al-Hazzaa, Abahussain, Al-Sobayel, Qahwaji, and Musaiger (2011) conducted a survey on the Saudi people in major cities in the KSA. They found the majority of Saudi adolescents spent more than 2 hours watching TV, and around half of them do not meet the daily physical activity requirements/standard (ibid). Around a quarter of them do not consume the recommended daily portions of milk, fruits and vegetables. Also, around a quarter of them have high daily intake of unhealthy food and drinks such as French fries, cakes and donuts, energy drinks and candy and chocolate (ibid).

Little research though was conducted in the KSA with regard to the effect of the built environment on diabetic patients whereas it is evident that urbanism and poor built environment conditions in the KSA (Khodeir et al. 2012) promote unhealthy lifestyles and aggravate diabetes (Sidawi & Hariri, 2012).

Diabetes affects the number and/or function of sweat glands, so it would decrease the volume and effective evaporation of sweat that is necessary to heat dissipation during a heat wave (Guyton & Hall, 2000). This can considerably increase the risk of hyperthermia and heatstroke (Kritikou-Pliota, Galanakis, Alfadaki, Siamopoulou, & Papadopoulou, 2000; Martinez, Devenport, & Saussy, 2002; WMO, 2010). Therefore, heat waves in hot climates would increase the risk of heat­related mortality such as diabetes, fluid/electrolyte disorders and some neurological disorders.

## 3. The Impact of Built Environment on Health

Barton et al. (2010) have classified the impact of the built environment on health into two categories: direct and indirect. Direct impacts include those traditionally associated with: a) planning and b) environmental health, e.g. air quality (indoor and outdoor), climate, water quantity and quality, noise and traffic-related injuries (Lavin, Higgins, Metcalfe and Jordan, 2006, Sustainable Development Commission, 2008). Indirect impacts include how the characteristics and design of the built environment influence the determinants of health, in particular perceptions of the local area, social connections and physical activity, which in turn are associated with physical and mental health and well-being (Barton et al., 2010). The section below discusses the home and neighborhood conditions and its’ impact on residents.

## 4. The Indirect Impacts of Built Environment

### 4.1 Home and Neighborhood Characteristics

This section discusses the characteristics of a home and neighborhood, such as its’ socio-economic status, how it promotes social connections, accessibility, physical activity, and whether it creates positive perception for the residents in their neighborhood.

At home level, a US study has looked at many risk factors for diabetes including physical characteristics and personal habits (e.g. weight, smoking, exercise, and alcohol use), marital status, education and housing conditions. The study rated the houses on the following basis: cleanliness inside of the building, the physical condition of the building’s interior and exterior, and the condition of the furnishings in the building. The researchers tested the impact of housing conditions and used physical attributes and habits as mediators and they found that housing conditions still influence diabetes risk and contribute to the development of diabetes (Schootman, Andresen, Wolinsky, 2006). For instance, dampness in buildings would critically affect health and a study found that damp housing is often associated with poor maintenance of the dwelling and socio-economic disadvantage of the occupants (Environmental Epidemiology Unit, 1999). There is a growing body of literature suggest that the progression of the Diabetes and its complications resulting in part from poor lifestyle habits such as lack of physical activity, which are in turn affected by built environment factors (Sudhir, Appa, & Sridhar, 2010).

It was known the influences of interactions between the level of activity and psychosocial wellbeing and stress in the pathogenesis of T2DM.1 (Sridhar et al., 2010). Furthermore, genome-wide association studies have identified the complex interplay between genes and the environmental factors that may change in genes expression making the genes potentially important pathogenic mechanisms in Diabetes and its consequences (Jirtle & Skinner, 2007).

Stewart et al. (2011) conducted a study in the USA that explored potential county-level associations between diabetes prevalence among adult African Americans and five socioeconomic characteristics: persistent poverty, unemployment, rurality, the number of fast food restaurants per capita, and the number of convenience stores per capita. They found diabetes prevalence rates in South Carolina where African American adults live, are among the highest in the nation, and there is a connection between the socioeconomic measures and diabetes. So, urban areas that have the above socioeconomic characteristics, would highly likely to have higher rates of diabetes prevalence (ibid).

Poorer health status in socioeconomically deprived and rural environments may reflect, in part, the inaccessibility of built environmental features such as public pools, recreation centers, physical fitness facilities, parks, sidewalks, and streetlights (Goldberg et al., 2000).

Allanah, Ashley and Farley (2010) stated that an ill designed urban setting would enforce people to adopt an unhealthy lifestyle thus contribute to the development of T2DM. Eckel et al. (2004) and Michaud et al. (2001) have linked passive entertainment and a lack of physical activity with the increasing incidence of the diabetes. Physical activity has been found to reduce the symptoms of coronary heart disease and strokes associated with T2DM (Frumkin, Frank, & Jackson, 2004). The decline in local facilities, the reduction in pedestrian movement and neighborly street life all reduce opportunities for the supportive social contact so vital for mental well-being (Barton et al., 2006). The absence of facilities, barriers to facilities (such as steep hills, busy roads to cross) or the perception that facilities are inadequate have negative associations with physical activity (Humpel, Owen, & Leslie, 2002).

In respect of social connections, researchers have linked fewer and weaker social networks with a number of health outcomes, including cardiovascular disease, mental health problems and increased mortality (Frumkin & Jackson, 2004; Balanda & Wilde, 2003).

So, neighborhood design that promote social networks are those which have mixed use and pedestrian oriented, with public spaces, such as parks, that can act as places for socializing (Leyden, 2003; Frumkin et al., 2004; Greenspace Scotland, 2008).

Those living in more “walkable” neighborhoods, characterized by high population density, mixed land use and high levels of connectivity (e.g. good pedestrian and cycling facilities) have been found to be more physically active (Handy, 2005; Saelens, Sallis, Frank, & Engelke, 2005, Frank, Kavage, & Litman, 2005, Pikora, Giles-Corti, Knuiman et al. 2005; Duncan, Spence, & Mummery, 2005).

#### 4.1.1 The Neighborhood Characteristics in Saudi Arabia

In the KSA, there are a number of spatial arrangements that can be defined according to the spatial configuration, such as gated compounds of different sizes and facilities, terraced housing, villas, blocks of flats, or mixed development. These also can be categorized according to socio-economic dimensions such as deprived and poor areas, middle class and high class areas. Khodeir *et al*. (2012) and Husain (2012) have highlighted the poor living conditions caused by heavy air pollution. The initiation of rapid urban growth of cities, particularly industrial cities causes substantial harm to the environment with industrial pollutants (Vincent, 2009). Allanah (2010) and Sidawi and Hariri (2012) found that such appalling living conditions would negatively affect the health of all citizens, including those with diabetes, and their perception of the neighborhoods. The irregular setting of community centres within their neighborhoods (Choguill, 2008) would mean that these centres are inaccessible to citizens. A recent pilot study in Dammam, Eastern province highlighted the opinions of diabetic patients who see that public gardens and sports centres are not well located within the urban context/fabric (Sidawi and Hariri, 2012). This would make it difficult for the patients to enjoy walking to and within these centers and parks particularly during the harsh hot weather that lasts around six months in the Eastern province, KSA. The results showed that the frequency of the symptoms is higher for patients who suffer more frequently from indoor/outdoor environmental/urban conditions. Patients who experience more frequently from drowsiness said that they have higher difficulty in wondering around within the neighborhood (ibid).

In addition, the extreme hot, dry and humid weather, in desert and coastal regions respectively, affects people’s type of activities and habits. As a result, Saudis perform alternative unhealthy activities such as adopting irregular sleeping patterns (e.g. afternoon nap, sleeping late), evening shopping trips to local malls and shopping arcades or sitting in coffee shops and these activities replace outdoor activities such as children’s playgrounds, and mothers walking children to school (Choguill, 2008). Also, a number of lifestyle issues should be appreciated as it would have an impact on the built environment (Sidawi and Meeran, 2011, and Sidawi, 2012) and health. These include the Islamic lifestyle presented by a number of aspects such as the five daily trips to the mosque to perform the five prayers, Ramadan Fasting, pilgrimage to Makkah etc and the Saudi culture represented by segregation between men and women, the regular male gatherings in the community centre, and the strong tribal and family relationships. In considering these parameters, there would be more chance of designing a humane and healthy built environment for Saudi citizens.

### 4.2 Perceptions of Place and Health

Many researchers have tackled the potential impact of neighborhood characteristics on people’s health (e.g. Kawachi & Berkman, 2003, Sampson, Morenoff, & Gannon-Rowley, 2002). The research mainly focuses on the overall neighborhood material conditions, illustrated through aggregate indices of deprivation or through measures of poverty, education and unemployment (Kobetz, Daniel, & Earp, 2003, Malmstro¨m, Johansson, & Sundquist, 2001; Stafford & Marmot, 2003; Wiggins et al., 2002). One way of doing this is to look at people’s perception of place, especially of problems and of social cohesion in the neighborhood, in relation to their health.

Perception of problems refers to aspects people say they dislike about their local area when interviewed in general health surveys (NICE, 2007). Such problems have been considered in relationship to health (Balfour & Kaplan, 2002; Latkin & Curry, 2003; Parkes & Kearns, 2005; Stafford & Marmot, 2003). Some problems can be labelled as physical or environmental when they relate to the presence of noise, dense traffic, dirt, odours, fumes or various signs of deterioration in the built environment such as abandoned buildings, trash, litter, graffiti and vacant housing (NICE, 2007). Other problems refer to the absence of basic infrastructure, facilities and amenities in the neighborhood (ibid).

Studies have consistently found evidence of a relationship between neighborhood environment (both perceptions and more objective measures) and self-reported health (Cummins et al., 2005; Curtice et al., 2005; Wilson et al., 2006). For example, people who perceive their neighborhoods to be hostile, dirty, poorly maintained, and lacking in safe places to play, are more likely to experience anxiety, depression, and poor health (Curtice et al., 2005).

Reported neighborhood problems and perceptions of neighborhood cohesion were both significantly associated with health outcomes after controlling four socio-demographic variables (Ellaway & Macintyre, 2001). This was especially true for the total number of symptoms and for the mental health scores (ibid). Whilst the stronger association found with psychosocial health variables seems entirely plausible, it should be noted that most studies of the relationship between health and income inequalities (or social capital), across nations or regions, have used mortality measures as the dependent variable (ibid).

Previous studies have shown the general level of participation in neighborhoods activities to be important for people’s health and not simply what the individual does him or herself (Ellaway & Macintyre, 2000).

On the other hand, high self-efficacy, perceptions of good quality facilities in the area and high levels of neighborliness were independently associated with good self-rated health and physical functioning. Perceptions of problems in the area were also predictive of poorer health (NICE, 2007).

A study on the residents of a number of neighborhoods in the Que´bec City region and it had tested the self- reporting of their physical conditions such as poor self-rated health, long-term disability, low feeling of mastery and their perception of neighborhood on health (Pampalona, Hamela, Koninckb, & Disant, 2007). The study found that after considering individual attributes, the perception of problems and social cohesion varies significantly by neighborhood and/or localities and can be considered as contextual variables. Furthermore, these perceptions of place appear to be significant predictors of people’s health (ibid).

The perception of problems in the neighborhood is negatively associated with several health outcomes such and health behaviors (NICE, 2007). Moreover, after accounting for the level of material deprivation of the neighborhood, significant relationships still remain between perceived problems and health outcomes (Steptoe & Feldman, 2001; Stafford & Marmot, 2003). Therefore, perceived problems and social cohesion in the neighborhood are related to people’s health and considered as mediating variables between neighborhood deprivation and health.

Such perceptions might influence people’s health through various pathways (Kawachi & Berkman, 2003; Parkes & Kearns, 2005). Perceived environmental problems, such as air or water pollution would affect physiological pathways, whereas perceived problems and social cohesion may influence health through psychosocial and physiological pathways (Latkin & Curry, 2003; Steptoe & Feldman, 2001). So, perceived problems in the neighborhood, such as noise and dense traffic are considered as chronic stressors heightening the level of anxiety, insecurity and fear among residents. Perceived local environments might influence health through behavioral pathways such as smoking, diet and physical activities).

The perception of social ties is also important. It is found that the role of shame, trust, social cohesion and more social capital (this term describes the social resources available to people through participation in civic and community networks, essential social infrastructures and organizations, connections and cohesiveness) are key determinants of health inequalities (Wilkinson, 1999). Health inequalities are the product of perceptions of relative income, producing negative emotions like shame and distrust which in turn are translated into poorer health at the individual level through psychoneuroendocrine mechanisms and/or health-damaging behaviors (Sapolsky, 1998). Consequently, when local social ties are seen as distant, unsupportive and mistrustful, collective strategies for fighting against or coping with local problems are less likely to emerge and, concurrently, the feeling of control residents might have over their own life may be reduced.

## 5. Healthy Built Environment and Healthy Lifestyle

The built environment should be designed to promote healthy lifestyles, facilitate social cohesion and offer access to educational, cultural, leisure and retail facilities capable of sustaining urban development (Bentivegna et al., 2002; Curwell & Deakin, 2002; Barton et al., 2010; Barton & Grant, 2011). To promote health for those with diabetes, the World Health Organization (WHO) set a number of healthy planning principles: healthy lifestyle, social cohesion, housing quality, access to employment and education opportunities, accessibility, local low-input food production, safety, equity, air quality and aesthetics, water sanitation and quality, quality of land and mineral resources, and climate stability (Barton & Tsourou, 2000).

Jackson and Kochtitzky (2001) advocated providing neighborhood opportunities for walking to accomplish routine activities such as shopping, going to work and exercise. Green areas should be carefully located, designed and integrated with the neighborhood in order to be pleasant in appearance, encourage walking and improve health. Landscape architecture appears to be the primary key at the finest scale to sound mind and body, and simply viewing nature reduces the stress of daily urban life (Ulrich, 1979; Jackson, 2011). Residents in more “walkable” neighborhoods, characterized by high population density, different types of land use, high connectivity (e.g. easy routes between destinations), good pedestrian and cycling facilities, and good accessibility, undertake more physical activity (Handy, 2005; Frank & Engelke, 2005; Pikora et al., 2005; Duncan, Spence, & Mummery, 2005). In addition, urban green space does more than offer opportunities for physical activity; it offers opportunities for engagement with, and observation of, nature, as well as opportunities for social interaction, thus enhancing individuals’ sense of well-being (Greenspace Scotland, 2008). Accessibility for all types of users to all facilities and services is a must. Visual landmarks and logical transit pathways assist people, particularly the elderly, in reaching their destinations. Psychologically, the above-mentioned elements provide a sense of ease and comfort.

On a neighborhood, urban or city level, urban planning plays a vital role in encouraging walking, the use of public transport, minimizing the number of vehicles on roads, providing well (cross) ventilated and shaded urban areas in hot climate countries, and exposure to sun for urban areas in cold countries. It also reduces the pollution and noise levels and would provide thermally comfortable urban areas.

## 6. The Research Objectives and Methodology

Considering the valuable contribution of the previous research in regards to the relation between the built environment and health, the aim of this research is to test the effect of the built environment on diabetic individuals within the context of Saudi Arabia.

Therefore, the researcher has set a number of objectives and these are:

- To find out the relation between the disease’s symptoms and resulted medical investigations of patients with the characteristics of their residence;
- To find out possible links between the disease’s symptoms and medical investigations of patients with their perception of the neighborhoods; and
- To make recommendations on how to enhance the Saudi built environment to be healthier.

Consent was firstly obtained from the University committee for biological and medical ethics. Thus, the field study was initiated in 2013 on diabetic individuals. The study’s sample constituted of patients who usually visit the diabetic clinic at the King Fahd teaching hospital of the University of Dammam, Al-Khobar. The choice of sample was based on the following criteria: the exclusion of severely ill, and children patients; and the inclusion of T1& 2DM male and female adults, age 15-70 years old and residents in the Eastern province, KSA.

Seventy six patients have participated in the study. These patients were handed a questionnaire form to fill in, and physical exams of patients’ health statuses were carried out. The questionnaire includes questions on the characteristics of their home and neighborhood, the patients’ lifestyles and activities since the onset of the disease, their perception of the environmental conditions of the home, work and neighborhood, and self-reported health conditions since the onset of the disease. All patients have filled in the questionnaire and returned it. The patients were physically examined. The exams include; a blood pressure test and Body Mass Index (BMI). The medical test results such as the fasting blood glucose, HBA1C, urine albumin, and lipid profile, were extracted from the patients’ medical files. The data collected from physical exams and the questionnaire survey was analyzed using SPSS.

## 7. The Research Findings

### 7.1 The Direct Results

The participants were asked about the distance between their homes and the local amenities. More than half of the respondents said that the public amenities are within walking distance i.e. up to 500 meters whereas 24% said that it is more than 1000 m from where they live. Around a quarter (23%) of the respondents said that the public garden is more 2000 m from their homes. 38% said that the recreation and sport center is more than 2000 m from their homes.

The respondents were asked about their daily life activities. More than half of the respondents (i.e. 52%, 57%) said they never or rarely do any recreational activities or morning sport exercises. 41% of the respondents said that they never or rarely do any social activities, 33% said that they never or rarely do any religious activities. Around half of them (48% - 59% respectively) said that rarely or never drink fizzy drinks or eat junk food meals. 86% of the respondents said that they never or rarely smoke. Around quarter of the respondents said that they rarely or never walk for 30 minutes whereas half (48%) of the respondents said they walk for 30 minutes daily. 72% of the respondents said that they often or always watch TV or work in the office. Around two third said that they often or always eat fruits and vegetables.

A quarter to one third (i.e. 24% to 34%) of the respondents have sometimes or often respectively, suffered from the following spatial and environmental conditions (table 1):

**Table 1:** How frequent a number of home and the neighborhood conditions are experienced by the diabetic individual since the onset of the disease (scale: 1 never to 5 always)

Home and the neighborhood conditions	Never or rarely (%)	Sometimes (%)	Often or always (%)
Low level of ventilation in my house	68	21	11
Annoying flow of air in my house	68	21	11
Uncomfortable temperature in my house	78	19	4
Lack of sunlight entry to the house	53	17	30
Poor air quality in the house	83	7	10
Unpleasant outside views	65	17	17
Disgusting odors in the neighborhood	80	17	3
lack of hygiene/cleansing in the neighborhood	79	14	7
Poor finishing of my house	79	17	3
The house organization is uncomfortable and it’s size is small	76	14	10
Uncomfortable furniture	86	10	3
Traffic noise	76	14	10
Noise from neighbors	73	10	17
Polluted environment of the residential neighborhood	85	15	0
Difficulty of wandering around in the neighborhood	73	20	7

- The house organization is uncomfortable and it’s size is small
- Low level of ventilation in the house
- Annoying flow of air in the house
- Unpleasant outside views
- Noise from neighbors
- Traffic noise
- Difficulty of wandering around in the neighborhood

The most frequent diabetes manifestations that are experienced by the respondents are: extreme fatigue/tiredness, tension/stress, blurred vision, and inability to control their nerves. Whereas the least experienced symptoms are: cardiatric problems, loss of sensation, loneliness and isolation and blood pressure problems.

### 7.2 The ANOVA Results

It should be mentioned that the study reports the significant results only (i.e. 0.5> P). The ANOVA results show that the house type (i.e. villa, duplex, flat or apartment) and area is associated with patients’ lifestyles and perception of home and neighborhood conditions. For instance, respondents who live in villas watch TV more frequently than those who live in flats duplexes or other types of dwellings. Respondents who live in flats or duplexes reported that they more frequently suffer from the lack of sunlight entry to their homes, poor air quality, unpleasant outside views, and noise from neighbors, than those who live in villas.

The ANOVA results show that the environmental conditions as experienced by the respondents at home are linked with the disease’s symptoms. Respondents, who suffer more frequently from the poor air quality in their houses, said that they experience Paresthesia symptoms more frequently.

Respondents, who more frequently experience uncomfortable furniture, said that they more frequently suffer from Paresthesia, blurred vision, tension/stress, or feeling overloaded. Respondents, who more frequently experience a low level of ventilation in their homes, said that they more frequently feel overloaded and an inability to relax.

Respondents, who more frequently experience an annoying flow of air in their homes, said that they more frequently suffer from a poor memory, and an inability to concentrate and relax. The uncomfortable temperature also causes a problem, as it constantly enforces the patient to sleep. Respondents who reported a poor finishing of their homes or said the organization of their houses is uncomfortable, said that they more frequently and constantly have blood pressure problems.

The study discovered an association between the actual measurement of medical test results and the home’s conditions. For instance, respondents who suffer more frequently from low level of ventilation in their homes, have higher lipid LDL (i.e. Low-Density Lipoproteins) and TG (i.e. triglyceride) levels. It is also found the lower/poorer lipid HDL (i.e. High-Density Lipoproteins) or unhealthy levels of lipid; are associated with uncomfortable internal furnishings.

On a neighborhood level, the study found an association between socio-economic conditions of the neighborhood and the individual diabetic’s lifestyle. Respondents who live in rich and upper middle areas tend to watch TV or work more in the office. On the other hand, respondents who live in poor areas suffer more from the following environmental conditions: poor air quality in the house, unpleasant outside views, noise from neighbors, polluted environment, and difficulty of wandering around in the neighborhood.

Respondents who reported poor condition of the neighborhood such as horrible odors and pollution said that they more frequently suffer from paresthesia, Blurred vision, and stress. The study found a relation between the onset of the disease and the location of the recreation and sport centers. Respondents, who had diabetes earlier, said that the recreation and sport centers are farther from their homes than those who had diabetes later.

## 8. Discussion and Recommendations

Many previous researches have pointed out to a number of links between the built environment and health. It proved that the place’s characteristics and people’s perceptions of the environmental and social conditions of their neighborhoods affected their health’s status through a number of pathways such as; psychosocial, physiological and behavioral pathways (Barton et al., 2010). This is true for diabetic patients who suffer from housing conditions, environmental pollution inside and outside their homes, socio-economic disadvantage of their neighborhoods, the inaccessibility of built environmental features such as recreation and sport centers, parks and walkways (see for instance Schootman, Andresen, & Wolinsky, 2006). This would force people to adopt unhealthy lifestyles, and contribute not only to the development of diabetes T2DM but to the deterioration of the health status of T1DM patients (Allanah, Ashley, & Farley, 2010; Goldberg et al., 2000; Eckel et al., 2004; Jirtle & Skinner, 2007).

In this paper the researchers examine the negative impact of the built environment on health of specific population that is the diabetic individuals in the KSA. The study shows that the progress of the disease is not only caused by an unhealthy lifestyle but also by an unhealthy built environment (Khodeir et al., 2012; Sidawi & Hariri, 2012). This unhealthy built environment in the KSA has certain characteristics: irregular setting of some of the community services such as public amenities, public garden and recreation and sport center within the neighborhoods. Previous research has highlighted also the high pollution levels, and irregularity in terms of spatial configuration/arrangement of features.

The survey found that such irregular settings of urban services would promote unhealthy lifestyles as around half of the respondents never or rarely do any recreational activities, morning sport exercises or participate in social activities whereas one third do religious activities (Barton et al., 2006; Humpel, Owen, & Leslie, 2002).

In regards to the dietary habits, around half of the respondents admitted that they sometimes or always, drink fizzy drinks or eat junk food meals whereas around three quarter said that they often to always watch TV or work in the office. This Obesogenic environment observed in our study could play an important role in the prevalence of the Diabetes and its consequences (Lake & Townshend, 2006)

Accordingly, around a quarter to one third said that they suffer sometimes or often from various home or neighborhood’s environmental conditions such as unpleasant outside views, noise from neighbors, traffic noise etc. Also, patients said they suffer mostly from extreme fatigue/tiredness, tension/stress, blurred vision, and an inability to control their nerves.

The study found significant links between the place i.e. home and neighborhood characteristics and the prevalence of diabetes. There is an association between the house type i.e. flat, duplex, villa, and the patients’ lifestyles and their perception of home and neighborhood conditions. Furthermore, there are links between the environmental conditions at home and the frequency of disease symptoms whereas bad environmental conditions are associated with higher frequency of self-reported symptoms (Schootman, Andresen, & Wolinsky 2006). The home conditions also have links with the medical investigations results whereas uncomfortable home conditions from environmental, social and psychological perspectives have a bad effect on diabetic individuals and causes higher levels of LDL, TG and poorer HDL.

The present study found that, the socio-economic conditions of the neighborhood (i.e. poor, lower middle class, upper middle class, rich) are linked with the individual diabetic’s lifestyle. The rich/high class environment encourages some bad habits such as watching TV or working longer hours at the office. On the other hand, respondents who live in poor areas suffer more from the poor environmental conditions at home and in their neighborhoods and subsequently they suffer more from a number of diabetes manifestations such as: Paresthesia, Blurred vision, and stress (also see Barton et al., 2010 for health& poor environment correlations).

So, in conclusion, control and prevention of Diabetes mellitus will require radical changes in the Saudi built environment to promote physical activity, improve psychological and physiological conditions of diabetic individuals. However, as diabetes is a lifelong disease, it would be affected by lifelong and ongoing changes in the life of individuals and the built environment. In the context of KSA, these would include the followings:

- As highlighted above, Saudi cities have unique planning, social, cultural, environmental and economic characteristics that should be considered. The fabric of the cities is rapidly changing due to the urban sprawl on macro level, changes on a neighborhood, and ongoing changes on macro level. The latter are undertaken by Saudi citizens on their dwellings;
- the Islamic lifestyle and lifelong practices and the Saudi culture as highlighted above, thus the ongoing impact of these issues on the built environment should be appreciated;
- the onging negative changes in the environment due to the increased levels of desertification, pollution etc. in the KSA and Gulf countries and climate change at regional and global level.

The consideration of these potential ongoing factors would enable researchers to develop better understanding to the eternal relation between the prevalence of diabetes and the built environment in the KSA.
